# Dimerization Activity of a Disordered N-Terminal Domain from *Drosophila* CLAMP Protein

**DOI:** 10.3390/ijms23073862

**Published:** 2022-03-31

**Authors:** Evgeniya Tikhonova, Sofia Mariasina, Olga Arkova, Oksana Maksimenko, Pavel Georgiev, Artem Bonchuk

**Affiliations:** 1 Department of the Control of Genetic Processes, Institute of Gene Biology, Russian Academy of Sciences, 119334 Moscow, Russia; fall2987@mail.ru; 2 Center for Magnetic Tomography and Spectroscopy, Faculty of Fundamental Medicine, M.V. Lomonosov Moscow State University, 119991 Moscow, Russia; sm1024sm@yandex.ru; 3 Center for Precision Genome Editing and Genetic Technologies for Biomedicine, Institute of Gene Biology, Russian Academy of Sciences, 119334 Moscow, Russia; forarkova@mail.ru (O.A.); maksog@mail.ru (O.M.)

**Keywords:** dosage compensation, NMR, dimerization, intrinsically disordered protein, small-angle X-ray scattering, C2H2 protein, architectural protein

## Abstract

In *Drosophila melanogaster*, CLAMP is an essential zinc-finger transcription factor that is involved in chromosome architecture and functions as an adaptor for the dosage compensation complex. Most of the known *Drosophila* architectural proteins have structural N-terminal homodimerization domains that facilitate distance interactions. Because CLAMP performs architectural functions, we tested its N-terminal region for the presence of a homodimerization domain. We used a yeast two-hybrid assay and biochemical studies to demonstrate that the adjacent N-terminal region between 46 and 86 amino acids is capable of forming homodimers. This region is conserved in CLAMP orthologs from most insects, except Hymenopterans. Biophysical techniques, including nuclear magnetic resonance (NMR) and small-angle X-ray scattering (SAXS), suggested that this domain lacks secondary structure and has features of intrinsically disordered regions despite the fact that the protein structure prediction algorithms suggested the presence of beta-sheets. The dimerization domain is essential for CLAMP functions in vivo because its deletion results in lethality. Thus, CLAMP is the second architectural protein after CTCF that contains an unstructured N-terminal dimerization domain.

## 1. Introduction

Multi-zinc-finger proteins comprise the largest family of eukaryotic DNA binding transcription factors [1,2]. In *Drosophila melanogaster*, more than 170 proteins have at least five clustered zinc fingers of the C2H2 type (C2H2 clusters) involved in highly specific recognition of long DNA motifs of 12–21 bp [1,2,3]. In addition to C2H2 clusters, such proteins (C2H2 proteins) often have additional domains involved in multimerization and protein–protein interactions [4]. It is assumed that these proteins play a key role in organizing chromosome architecture and facilitating highly specific interactions between regulatory elements at large distances [5,6]. More than half of *D. melanogaster* C2H2 proteins have an N-terminal domain called the zinc-finger-associated domain (ZAD), which predominantly forms homodimers [7]. Interestingly, in mammals, only a small proportion of C2H2 proteins have structured N-terminal dimerization domains, such as BTB and SCAN [4,8,9,10,11].

An unusual multimerization domain has been found at the N-termini of CTCF proteins in various animal species [12,13]. CTCF is the most conserved C2H2 protein among higher eukaryotes and is believed to be the major architectural protein in mammals [5,14]. This domain lacks secondary structure and has biochemical properties more typical of an intrinsically disordered region (IDR) [12]. IDRs are abundant in chromatin-associated proteins [15,16], playing diverse roles in molecular recognition, chromatin compaction, and subcellular compartmentalization through phase separation [17,18].

CLAMP is a zinc-finger transcription factor that was originally discovered as a recruiter of a dosage compensation complex (DCC) on the male X chromosome in *D. melanogaster* [19,20]. DCC consists of five proteins (MSL1, MSL2, MSL3, MOF, and MLE) and the non-coding RNA components roX1 and roX2 [21,22]. The N-terminal zinc-finger C2H2 domain of CLAMP interacts with the unstructured region of the MSL2 protein [23]. According to modern concepts, the specific binding of a DCC to certain regulatory elements on the X chromosome of males is determined by the DNA-binding activity of MSL2, roX RNAs (whose role is not fully understood), and the specific interaction between MSL2 and CLAMP [23,24,25,26,27].

Growing evidence suggests that CLAMP has multiple functions beyond dosage compensation. CLAMP is an essential transcription factor that binds thousands of sites throughout the genome [28,29,30]. Similar to GAF and Zelda proteins, CLAMP is involved in zygotic gene activation (ZGA), a dramatic reprogramming that occurs in the zygotic nucleus to initiate global transcription and prepare the embryo for further development [31,32]. CLAMP and GAF are components of the late boundary complex (LBC), which binds the Fab-7 and Fab-8 boundaries in the bithorax complex and is involved in the regulation of long-distance interactions between enhancers and promoters [33,34,35,36]. CLAMP is associated with several transcription factors [28,29] and is involved in the regulation of Su(Hw)-dependent insulators [37].

Recently, CLAMP was implicated in chromatin architectural function, bridging distant genomic loci together [38]. Such an architectural function is frequently associated with N-terminal multimerization domains [39,40].

In this work, we investigated whether CLAMP has an N-terminal homodimerization domain similar to well-described architectural proteins. Using the yeast two-hybrid (Y2H) system, we mapped the dimerization domain between 46 and 86 amino acids of CLAMP. Studies using nuclear magnetic resonance (NMR) and small-angle X-ray scattering (SAXS) suggested that this domain is intrinsically disordered whereas the presence of beta-sheets was bioinformatically predicted. Dimerization was demonstrated to be essential for CLAMP functions in vivo. Thus, CLAMP has a disordered multimerization domain similar to the highly conserved CTCF, which is the main well-characterized architectural protein in mammals [13].

## 2. Results

### 2.1. N-Terminal Domain of CLAMP Protein Forms Dimers

**Figure 1:**
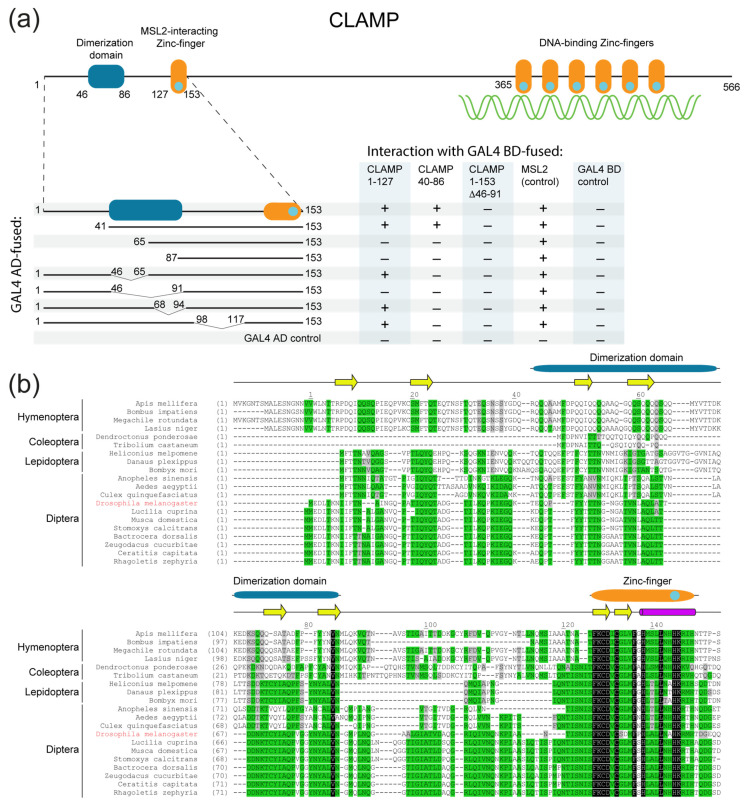
(**a**) Results of the Y2H assay of dimerization activity of the CLAMP N-terminal region shown for the domain structure of the CLAMP protein. AD stands for GAL4 activation domain and BD stands for GAL4 DNA-binding domain. Positive interaction indicates the ability of yeast to grow on assay plates without histidine. Assay plates are shown in Figure S2. (**b**) Multiple sequence alignment of N-terminal domains of CLAMP proteins from various insects performed with ClustalW [47]. The positions of predicted secondary structure, dimerization, and zinc-finger domains are shown. Residue numbering corresponds to *D. melanogaster* CLAMP.

The CLAMP protein of *D. melanogaster* (Figure 1a) contains a C-terminal cluster consisting of six C2H2 domains that are responsible for binding to the (GA)n motif [20]. The N-terminal region includes a single C2H2 domain that is highly conserved in insects (Figure 1b). Previously we found that the 40–153 region of CLAMP, including the C2H2 domain, binds MSL2 [23]. Except for the C2H2 domain, N-terminal sequences contain few sequence blocks that display modest conservation (Figure 1b) and are predicted to form beta-sheets (Figure S1) [41,42,43,44,45].

We recently found that the region of 1–153 amino acid residues can homodimerize in the Y2H assay [46]. For detailed mapping of the CLAMP domain involved in homodimerization, we tested different variants of the N-terminal region for interaction in the Y2H assay. The deletion of the C2H2 domain in the N-terminal sequences of CLAMP (CLAMP^1–127^) did not affect interaction with CLAMP^1–153^, suggesting that the C2H2 domain is not essential for homodimerization (Figure 1a). Using deletion derivatives, we further mapped the minimal sequence required for dimerization in the 46–86 region (Figure 1a and Figure S2). Interestingly, deletions of either 46–65 or 68–94 amino acids weaken but did not disrupt dimerization (Figure S2); therefore, both its first and second 20 residues are sufficient for the interaction, suggesting the lack of distinct spatial folding of the domain. All deletion derivatives containing intact zinc-finger bound efficiently to MSL2, confirming the correct folding of the domain (Figure 1a).

**Figure 2:**
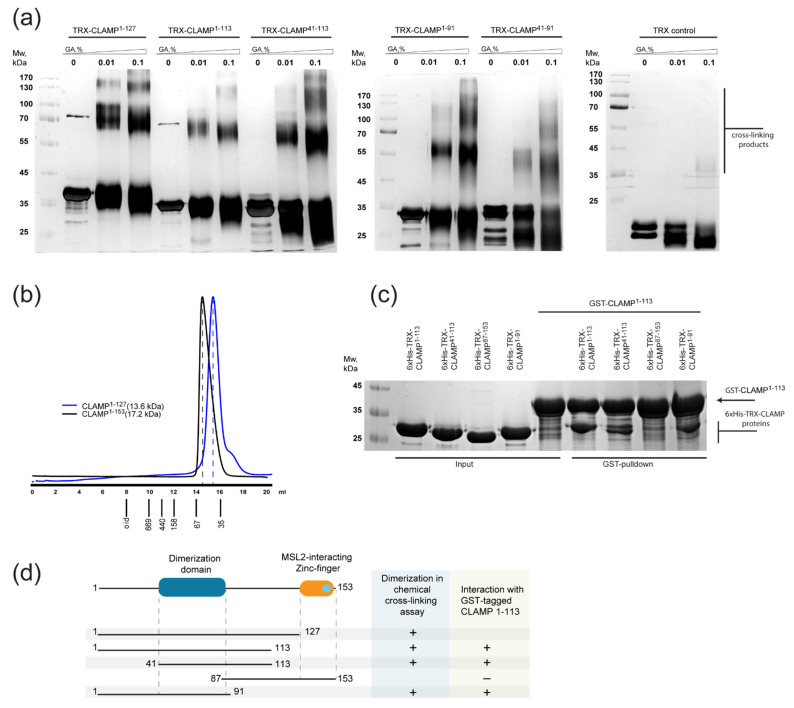
(**a**) Cross-linking of thioredoxin-tagged CLAMP derivatives using increasing concentrations of glutaraldehyde (GA). Uncropped images are shown in Figure S3. (**b**) Superdex S200 size-exclusion chromatography of CLAMP ^1–127^ and CLAMP^1–153^. Molecular weights of the monomers are shown in brackets. (**c**) Testing of the dimerization specificity of CLAMP deletion derivatives in glutathione S-transferase (GST). CLAMP derivatives fused either with GST or with 6xHis-thioredoxin were co-expressed in bacteria cells and affinity-purified with glutathione resin (which binds GST-tagged proteins). 6xHis pull-down assays are shown in Figure S4. Co-purified proteins were visualized with SDS-PAGE followed by Coomassie staining. Uncropped images are shown in Figure S4. (**d**) Summary of dimeric interactions observed with pull-down and cross-linking assays.

Then, CLAMP deletion derivatives were expressed in bacteria and purified for subsequent biochemical studies in vitro. Using chemical cross-linking (Figure 2a,c) and size-exclusion chromatography (Figure 2b) experiments, we demonstrated that the CLAMP^1–127^ can form multimers (presumably dimers) similar to CLAMP^1–153^. Shortening of the region to 1–113 amino acids and deleting the first 40 residues only weakly reduced the efficiency of cross-linking, supporting the data of Y2H (Figure 2a). Further shortening of the region decreased the cross-linking efficiency (Figure 2a and Figure S3), likely because cross-linking is dependent on the presence of neighbor lysines; thus, it is not suitable for the precise mapping of the dimerization motif and was not used on smaller fragments. Interactions between 6xHis thioredoxin- or glutathione S-transferase (GST)-tagged CLAMP deletion derivatives were further studied with a pull-down assay after co-expression in bacteria cells (Figure 2c and Figure S4). Because CLAMP^1–113^ binds non-specifically to Ni-NTA resin, 6xHis pull-down was used only as protein expression control. CLAMP^41–113^ interacts efficiently with the larger GST-tagged CLAMP^1–113^ polypeptide; however, the interaction between CLAMP^1–91^ and CLAMP^1–113^ polypeptides was slightly impaired. The 87–153 fragment lacking most of the dimerization sequences did not interact with CLAMP^1–113^. Taken together, these results suggest that the 46–86 amino acids are sufficient for dimerization and include two modules that can both form dimers. Interestingly, these regions coincide with the conserved sequences that were predicted to form beta-sheets.

To further assess the folding state of the domain and measure the molecular weights of multimers, we applied the small-angle X-ray scattering (SAXS) technique to the deletion derivatives of CLAMP (1–153, 1–113, and 87–153 amino acids) in solution. SAXS provides precise information about the size of macromolecules in solution that is almost independent of their shape [48]. Molecular weight estimation of CLAMP^1–153^ using extrapolated I_0_ scattering intensity yielded a value of 24–30 kDa at a concentration of 2.3 mg/mL, which is in agreement with dimer formation; however, increasing the sample concentration to 11.8 mg/mL resulted in the doubling of that value (60–75 kDa), suggesting tetramerization, but this is likely an effect of higher-order low-specific association due to a high sample concentration (Table 1). By contrast, the molecular weight estimation for CLAMP^87–153^ resulted in values in the range 5.1–6.8 kDa, which corresponds to the monomer. The estimated molecular weight CLAMP^1–113^ falls into the range of 20–24 kDa, which confirms that it exists as a dimer in solution.

### 2.2. N-Terminal Domain of CLAMP Protein Is Disordered

**Figure 3:**
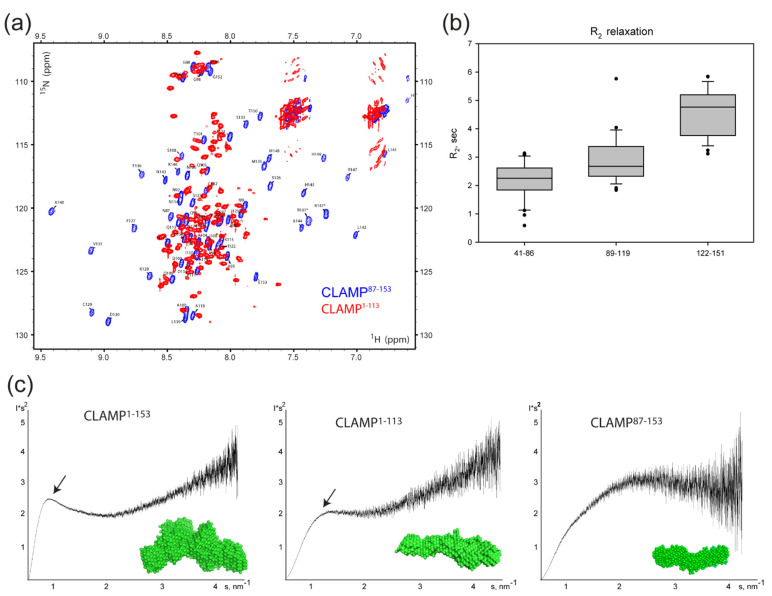
(**a**) ^15^N-^1^H-HSQC spectra of *D. melanogaster* CLAMP^1–113^ (red) and CLAMP^87–153^ (blue). The amino acid assignment (performed for residues 87–153 in [46], BioMagResBank ID: 34600) is shown. (**b**) R_2_ NMR relaxation times for CLAMP residues in 41–86, 89–119, and 122–151 regions. (**c**) Kratky plots (I*s2 vs. s) of SAXS data derived for CLAMP^1–153^, CLAMP^1–113^, and CLAMP^87–153^ to assess the folding state of protein molecules according to [51]. Bell-shaped areas indicative of folded regions are shown with arrowheads. Averaged ab initio bead models developed from SAXS data are shown as green surfaces (calculated from data obtained at 2.3 mg/mL (CLAMP^1–153^), 7.0 mg/mL (CLAMP^1–113^), and 10 mg/mL (CLAMP^87–153^) by the DAMMIN shape reconstruction program [52] and averaged with the DAMAVER algorithm [53]).

To assess the folding state of the CLAMP N-terminal domain, we used NMR spectroscopy. We measured the ^15^N-HSQC NMR spectrum of ^15^N-labeled CLAMP^1–113^ and compared it with a spectrum of CLAMP^87–153^ including the zinc-finger region for which the resonance assignments were obtained previously ([46], BioMagResBank ID: 34600 Available online: https://bmrb.io/data_library/summary/index.php?bmrbId=34600 (accessed on 26 December 2021). All H_N_ chemical shifts of the 1–86 region are plotted into the interval from 7.6 to 8.6 ppm. These chemical shift values correspond to the unstructured protein chain [49] (Figure 3a). Notably, disorder prediction algorithms do not confidently predict the presence of disorder within this fragment (Figure S1c,d). To ensure that CLAMP^41−86^ is unstructured, we obtained ^15^N-labeled CLAMP^41−153^ and compared its HSQC spectrum with CLAMP^87−153^. As most signals in 87–153 are still present at their places in the spectrum of CLAMP^40−153^, we can assign the rest of the signals to the 41–86 region (Figure S5). In total, 38 peaks were found corresponding to that region (most probably several of them represent more than one signal because of peak overlapping). All H_N_ chemical shifts of the 41–86 region also fall into the interval from 7.6 to 8.6 ppm, suggesting a lack of the secondary structure [49]. Additionally, we measured transverse relaxation rates (R_2_) to assess the protein chain mobility in different regions. R_2_ reflects protein chain mobility and can be used to measure disordered state of the protein [50]. The R_2_ values are smaller for the unstructured protein chain. We measured R_2_ for the CLAMP^41−153^ sample and calculated its average values for residues in 41–86, 89–119, and 122–151 regions (Figure 3b). The averaged R_2_ value for 41–86 is 2.2 ± 1.3 s, whereas for 89–119 (unfolded region preceding the zinc-finger) it is 2.9 ± 0.8 s, and 4.6 ± 0.8 s for 122–151 (zinc-finger domain). Thus, we conclude that according to NMR data the CLAMP^41−86^ region is unstructured.

The Kratky plot of SAXS data (I*s^2^ vs. s) is useful for assessing the folding state of protein molecules [51]. The presence of a bell-shaped area indicates the presence of folded regions, whereas a log-shaped curve is more characteristic of disordered protein chains. The Kratky plot of CLAMP^1–153^ suggested the presence of a small proportion of folded regions, likely mostly within the zinc-finger domain (Figure 3c), which is in agreement with the NMR data and Kratky plot of CLAMP^87–153^ demonstrating a bell-shaped scattering profile. The same plot of CLAMP^1–113^ only revealed the presence of a small bell-shaped area, suggesting that it does not represent a completely unfolded polypeptide chain but rather lacks a stable spatial structure (Figure 3c). Several low-resolution models were built using the DAMMIN algorithm [52] on the basis of the scattering data of CLAMP^1–153^; the model averaged with DAMAVER is shown in Figure 3c. The overall shape also suggests the presence of two-fold symmetry.

Thus, the NMR and SAXS data suggest that the N-terminal domain of CLAMP preceding the zinc-finger has the features of an IDR.

### 2.3. N-Terminal Domain of CLAMP Protein from Apis mellifera Is a Monomer

**Figure 4:**
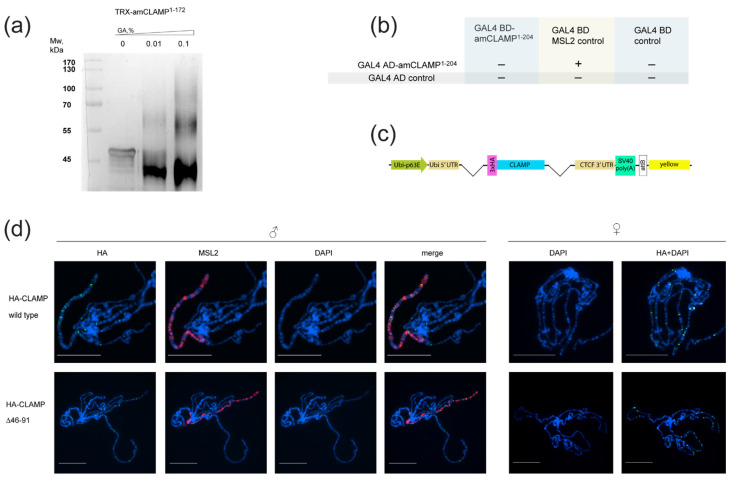
(**a**) Cross-linking of thioredoxin-tagged amCLAMP^1–172^ using increasing concentrations of glutaraldehyde (GA). (**b**) Testing of the dimerization activity of amCLAMP^1–204^ using a yeast two-hybrid assay. AD stands for GAL4 activation domain and BD stands for GAL4 DNA-binding domain. Positive interaction indicates the ability of yeast to grow on assay plates without histidine. Assay plates are shown in the Figure S2. (**c**) Schematic representation of rescue constructs expressing 3xHA-tagged CLAMP proteins under the control of ubiquitin-p63E promoter; SV40 poly(A)–SV40 polyadenylation signal; attB is the site for φC31-mediated recombination used for site-specific insertion of the construct; yellow represents the intronless yellow gene used as a reporter. (**d**) Effect of deletion of the dimerization domain in the HA-tagged CLAMP proteins on their recruitment to the chromatin shown by immunostaining of polytene chromosomes with anti-HA and anti-MSL2 antibodies in males and females. Scale bar is 20 μm.

Multiple sequence alignment showed that the CLAMP N-terminal zinc-finger is highly conserved, even in Hymenopterans (Figure 1b), whereas sequences preceding the zinc-finger domain are conserved in most insects except Hymenopterans (Figure 1b). To test whether CLAMP orthologs in Hymenopterans have unrelated N-terminal homodimerization domains, we examined the same region of CLAMP from the honey bee (amCLAMP). The amCLAMP^1–204^ includes the C2H2 zinc-finger domain, whereas amCLAMP^1–172^ does not. The longer construct should be able to interact with the *D. melanogaster* MSL2 protein as a control; thus, it was used in the Y2H assay. According to chemical cross-linking, amCLAMP^1–172^ is a monomer in solution (Figure 4a). In the Y2H assay, amCLAMP^1–204^ interacted with *D. melanogaster* MSL2 but did not interact with its counterpart (Figure 4b). These results indicate the absence of a homodimerization domain in the N-terminal portion of amCLAMP.

### 2.4. Integrity of Dimerization Activity of CLAMP N-Terminal Domain Is Essential for Its Functions In Vivo

To assess the effect of deletion disrupting the CLAMP dimerization in vivo, we obtained transgenic flies expressing 3xHA-tagged CLAMP^WT^ or CLAMP^Δ41–91^ under the control of the strong ubiquitin (*Ubi63E*) promoter (Figure 4c). Both transgenes were inserted into the same 86Fb region on the third chromosome using a φC31 integrase-based integration system [54]. We examined the ability of transgenes expressing wild-type and mutant proteins to complement the *clamp*^2^ mutation [55]. Expression of the CLAMP^WT^ protein restored the survival rate of *clamp*^2^ flies to a greater extent. Furthermore, both males and females homozygous for *clamp*^2^ expressing CLAMP^Δ41–91^ died at the late larvae (L3), similar to the *clamp*^2^ mutant alone. Thus, the N-terminal dimerization domain is essential for the general function of CLAMP. We compared the binding of CLAMP^WT^ and CLAMP^Δ41–91^ to polytene chromosomes from salivary glands (Figure 4d). Because no significant differences in binding between CLAMP^WT^ and CLAMP^Δ41–91^ were observed, we suggest that both proteins are expressed at the same level, and the N-terminal dimerization domain is not essential for CLAMP recruitment to chromatin.

## 3. Discussion

Here, we demonstrated that the N-terminal domain of the CLAMP protein is capable of forming dimers. The prediction algorithms suggested the presence of beta-sheets in this domain. At the same time, all used biophysical techniques, including nuclear magnetic resonance (NMR) and small-angle X-ray scattering (SAXS), suggested that the N-terminal domain has features of an intrinsically disordered region. In this way, CLAMP is similar to CTCF, which has an N-terminal unstructured domain that is involved in homodimerization [12]. As in the case of CTCF [12,13], the N-terminal domain is critical for the functional activity of CLAMP.

In comparison with CTCF, the N-terminal domain of CLAMP apparently has more dynamic folding, which is reflected by SAXS and NMR data more typical of IDRs. Intrinsically disordered proteins are abundant within the structures of transcription factors and other chromatin-associated proteins [15,18]. IDRs’ protein–protein interaction properties are difficult to predict [56], and thus similar domains could be widespread within eukaryotic transcription factors. Disordered regions provide a higher degree of dynamics and plasticity for protein function, which might be beneficial in the assembly of different regulatory complexes in the context of variable chromatin, as was described for p53 and calmodulin [57,58,59].

Tikhonova et al. [46] demonstrated that the N-terminal dimerization domain facilitates a relatively weak interaction between the C2H2 domain of CLAMP and MSL2. Because CLAMP exhibits architectural properties [38], the dimerization domain might be involved in the organization of distance interactions between regulatory elements in a manner similar to the CTCF protein. The dimerization through the disordered domain may create a malleable scaffold involved in the interaction with various proteins.

Previously, we found that the N-terminal IDRs of CTCF proteins obtained from different bilateral organisms do not have sequence homology but are capable of homodimerization [12]. By contrast, the N-terminal dimerization domain of CLAMP is conserved in most insects, except for Hymenopterans. CLAMP can be involved in the formation of chromatin loops that facilitate the spreading of DCC along the *D. melanogaster* X chromosome [38]. In honey bees, CLAMP does not play a role in dosage compensation and its N-terminal region lacks dimerization activity. It can be hypothesized that the N-terminal homodimerization domain may be required for the architectural function of CLAMP, which is essential for the spreading of DCC.

## 4. Materials and Methods

### 4.1. Plasmids and Cloning

cDNAs were PCR-amplified using corresponding primers (Table S1) and cloned into a modified pGEX4T1 vector (Cytiva, Marlborough, USA) encoding the TEV protease cleavage site after GST and into the vector derived from pACYC and pET28a(+) (Merck KGaA, Darmstadt, Germany) bearing a p15A replication origin, kanamycin resistance gene, and pET28a(+) MCS. *Apis mellifera* cDNA was prepared using standard procedures from adult bees obtained from a local apiary. For Y2H assays, cDNAs were amplified using the corresponding primers (Table S1) and fused with the DNA-binding or activation domain of GAL4 in the corresponding pGBT9 and pGAD424 vectors (Clontech, San Jose, CA, USA). Details of assembling the constructs for expressing proteins in transgenic flies are available upon request.

### 4.2. Yeast Two-Hybrid Assay (Y2H)

The Y2H assay was performed as previously described [40]. Briefly, for growth assays, plasmids were transformed into the yeast strain pJ69-4A by the lithium acetate method following the standard Clontech protocol and plated on media without tryptophan and leucine. After two days of growth at 30 °C, the cells were plated on selective media without tryptophan, leucine, histidine, or adenine, and their growth was compared after 2–3 days. Each assay was repeated three times.

### 4.3. Fly Crosses, Transgenic Lines, and Polytene Chromosome Staining

*D. melanogaster* strains were grown at 25 °C under standard culture conditions. The transgenic constructs were injected into preblastoderm embryos using the φC31-mediated site-specific integration system at locus 86Fb [54]. The emerging adults were crossed with the *y ac w*^1118^ flies, and the progeny carrying the transgene in the 86Fb region were identified by a *y^+^* pigmented cuticle. To assess the viability of transgenic lines expressing CLAMP^Δ40–91^, virgin *clamp*^2^/CyO, GFP; Ubi:CLAMP^Δ40–91^-HA/Ubi:CLAMP^Δ40–91^-HA females were crossed with *clamp*^2^/CyO, GFP; Ubi:CLAMP^Δ40–91^-HA/Ubi:CLAMP^Δ40–91^-HA males. The viability of transgenic flies expressing CLAMP^Δ40–91^ was calculated as the ratio of the homozygous males or females (*clamp*^2^*/clamp*^2^; Ubi:CLAMP^Δ40–91^-HA/Ubi:CLAMP^Δ40–91^-HA) relative to heterozygous males or females (*clamp*^2^/CyO; Ubi:CLAMP^Δ40–91^-HA/Ubi:CLAMP^Δ40–91^-HA) divided by two. Polytene chromosome staining and immunoblotting assay were performed as described in [23].

### 4.4. Protein Expression and Purification

BL21(DE3) cells transformed with CLAMP constructs fused with TEV-cleavable 6xHis-Thioredoxin were grown in 1 L of LB media to an A600 of 1.0 at 37 °C and then induced with 1 mM IPTG at 18 °C overnight. Cells were disrupted with high-pressure homogenizer (Microfluidics, Westwood, USA) in buffer A (30 mM HEPES (pH 7.5), 400 mM NaCl, 5 mM β-mercaptoethanol, 5% glycerol, 0.1% NP40, 10 mM imidazole) containing 1 mM PMSF and Calbiochem Complete Protease Inhibitor Cocktail VII (1 μL/mL). After centrifugation, lysate was applied to a Ni-NTA column, and after washing with buffer B (30 mM HEPES (pH 7.5), 400 mM NaCl, 5 mM β-mercaptoethanol, 30 mM imidazole) was eluted with 300 mM imidazole. For cleavage of the 6x-His-thioredoxin-tag, 6x-His-tagged TEV protease was added at a molar ratio of 1:50 directly to the eluted protein and the mixture was incubated for 2 h at room temperature, then dialyzed against buffer A without NP-40 and applied to a Ni-NTA column. Flow-through was collected; dialyzed against 20 mM Tris-HCl (pH 7.4), 50 mM NaCl, and 1 mM DTT; and then applied to a SOURCE15Q 4.6/100 column (Cytiva, Marlborough, USA). Fractions containing proteins were collected, concentrated, frozen in liquid nitrogen, and stored at −70 °C. Stable isotope-labeled proteins were expressed according to [60] and purified using the same procedures as those used for native proteins.

### 4.5. NMR Spectroscopy

The NMR samples contained 0.2 mM ^15^N-CLAMP^1–113^, 20 mM sodium phosphate at pH 7, and 5% (*v*/*v*) D_2_O for frequency lock. Two-dimensional NMR spectra were collected using the sfhmqcf3gpph pulse program on a Bruker (Billerica, MA, USA) AVANCE 600 MHz spectrometer equipped with a TXI triple resonance (^1^H,^13^C,^15^N) probe at 25 °C. 

### 4.6. Pull-Down Assays and Chemical Crosslinking

GST pull-down was performed with Immobilized Glutathione Agarose (Thermo Fisher Scientific, Waltham, MA, USA) in buffer C (20 mM Tris (pH 7.5); 150 mM NaCl; 10 mM MgCl_2_; 0.1 mM ZnCl_2_; 0.1% NP40; 10% [*w*/*w*] glycerol; 1 mM DTT). BL21 cells co-transformed with plasmids expressing GST-fused and 6xHis-Thioredoxin-fused derivatives of CLAMP were grown in LB media to an A600 of 1.0 at 37 °C and then induced with 1 mM IPTG at 18 °C overnight. ZnCl_2_ was added to a final concentration 100 μM before induction. Cells were disrupted by sonication in 1 mL of buffer C, and after centrifugation, lysate was applied to pre-equilibrated resin for 10 min at 4 °C; subsequently, resin was washed four times with 1 mL of buffer C containing 500 mM NaCl and bound proteins were eluted with 50 mM reduced glutathione, 100 mM Tris (pH 8.0), and 100 mM NaCl for 15 min. 6xHis pull-down was performed similarly, with Ni-NTA HP resin (Cytiva, Marlborough, MA, USA) in buffer A (see protein expression and purification section) containing 1 mM PMSF and Calbiochem Complete Protease Inhibitor Cocktail VII (5 μL/mL) and washed with buffer A containing 30 mM imidazole. Proteins were eluted with buffer B containing 300 mM imidazole (20 min at 4 °C). Chemical crosslinking was carried out for 10 min at room temperature in 20 mM HEPES (pH 7.7); 150 mM NaCl; 20 mM imidazole; and 1 mM β-mercaptoethanol. Prior to crosslinking, protein concentration was adjusted to 5 μM for at least 1 h. Crosslinking was quenched with 50 mM Tris-HCl (pH 6.8) and samples were resolved using SDS-PAGE followed by silver staining.

### 4.7. SAXS Data Collection and Analysis

Synchrotron radiation X-ray scattering data were collected using the standard procedures on the BM29 BioSAXS beamline at the European Synchrotron Radiation Facility (ESRF) (Grenoble, France) as described previously [12]. Data analysis was performed using the ATSAS software package [61]. Ab initio modeling was performed with DAMMIN [52], and an averaged model was calculated with DAMAVER [53].

## 5. Conclusions

In conclusion, we demonstrated the presence of an unusual dimerization domain at the N-terminus of the CLAMP protein. Despite being predicted to have a beta-sheet secondary structure, this domain has features of IDRs and is crucial for CLAMP functions. In comparison with typical multimerization domains with distinct spatial folding, disordered domains might provide more plasticity, allowing a wide variety of interactions in the assembly and control of the activity of genomic regulatory elements. 

## Figures and Tables

**Table 1:** Scattering parameters of CLAMP N-terminal domain derivatives. R_g_, radius of gyration (average of square center-of-mass distances in the molecule); D_max_, maximum dimension of the particles; V_p_, Porod volume (excluded volume of hydrated particles).

Protein	Sample Concentration, mg/mL	R_g_, nm	D_max_, nm	V_p_, nm^3^	Monomer Mw, kDa	Estimated Mw, kDa
CLAMP^1–153^	2.3	2.9	10.2	42.9	17	24–30
	11.8	3.9	13.6	120.9	17	60–75
CLAMP^87–153^	2.5	2.2	8.4	10.9	7.3	6.0–6.8
	10.0	2.3	10.9	9.4	7.3	5.1–5.9
CLAMP^1–113^	1.0	2.4	11.3	33.8	12.3	19–23
	7.0	2.7	11.9	36.3	12.3	20–24

## Data Availability

Not applicable.

## Supplementary Materials

The following supporting information can be downloaded at: https://www.mdpi.com/article/10.3390/ijms23073862/s1.
