# Supplementary material for: Dimerization Activity of a Disordered N-Terminal Domain from Drosophila CLAMP Protein
Source: Int J Mol Sci. 2022 Mar 31;23(7):3862. doi: 10.3390/ijms23073862 (PMC8998743; doi:10.3390/ijms23073862)

Supplementary information for:

Dimerization activity of disordered N-terminal domain from *Drosophila* CLAMP protein

Evgeniya Tikhonova[1], Sofia Mariasina[2], Olga Arkova[3], Oksana Maksimenko[3], Pavel Georgiev[1*], Artem Bonchuk[1,3*]

1 Department of the Control of Genetic Processes, Institute of Gene Biology Russian Academy of Sciences, 34/5 Vavilov St., Moscow 119334, Russia; fall2987@mail.ru (E.T.); georgiev_p@mail.ru (P.G.); bonchuk_a@genebiology.ru (A.B.)

2 Center for Magnetic Tomography and Spectroscopy, Faculty of Fundamental Medicine, M.V. Lomonosov Moscow State University, Moscow, 119991, Russia, sm1024sm@yandex.ru (S.M.)

3 Center for Precision Genome Editing and Genetic Technologies for Biomedicine, Institute of Gene Biology, Russian Academy of Sciences, 34/5 Vavilov St., Moscow 119334, Russia; forarkova@mail.ru (O.A.); maksog@mail.ru (O.M.)

* Correspondence to Artem Bonchuk and Pavel Georgiev: georgiev_p@mail.ru (P.G.) bonchuk_a@genebiology.ru (A.B.)

Supplementary Table S1. Oligonucleotides used for cloning. Restriction enzyme sites are shown in small letters, the corresponding enzymes are noted. Nucleotide substitutions in mutagenic primers are also shown in small letters.

	Direct	Reverse	REs
CLAMP $^{87-153}$	CTGgaattcATGGAAGACCTTACCAA	AACgtcgacTTCCCCGTCTGTATGCAT	*Hinc*II,*Sal*I
CLAMP $^{40-153}$	CTGgaattcATGAAAACGGAGCAGCAGC	AACgtcgacTTCCCCGTCTGTATGCAT	*Eco*RI,*Sal*I
MSL2	CGAGTACTGGTGGCGTGGGTCGGACCGAC	TCTgtcgacCAAGTCATCCGAGCCCGACA	*Sca*I, *Sal*I
CLAMP $^{1-153}$	CTGgaattcATGGAAGACCTTACCAA	AACgtcgacTTCCCCGTCTGTATGCAT	*Eco*RI,*Sal*I
HA-CLAMP	CTGgaattcATGGAAGACCTTACCAA	TAGgtcgacCTATAACCCACCGATAATC	*Eco*RI,*Sal*I
amCLAMP $^{1-204}$	GACggatccATGGTCAAAGGCAACACATC	TTGgtcgacTTATTGAAGATTACTAGGTGTTGTATTATG	*Bam*HI, *Sal*I
amCLAMP $^{1-172}$	GACggatccATGGTCAAAGGCAACACATC	TTGgtcgacCGCATTGGTAGCAGCCGC	*Bam*HI, *Sal*I

Supplementary Figure S1. Secondary structure prediction of CLAMP1-153 with (A) PsiPred [57], (B) QUARK server [58,59], (C) Intrinsic disorder prediction with IUPred2A [60]. (D) Disorder prediction with DISOPRED [61].

A

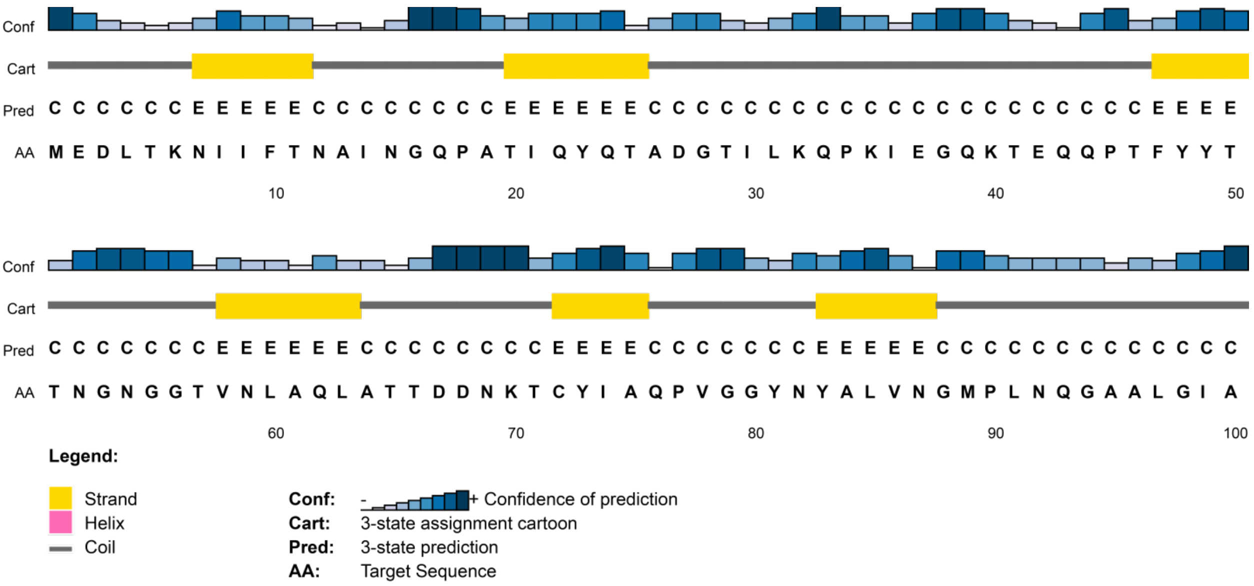

B

```
           1                  20                  40                  60                  80                  100
           |                   |                   |                   |                   |                   |
Sequence   MEDLTKNIIFTNAINGQPATIQYQTADGTILKQPKIEGQKTEQQPTFYYTTNGNGGTVNLAQLATTDDNKTCYIAQPVGGYNYALVNGMPLNQGAALGIA
Prediction CCCCCCCSSSSSCCCCCSSSSSSSSCCCCHHCCCSSCCSSCCCCCCSSSSSCCCCCSSSHHHHCCCCCCCSSSSSCCCCCCCSSSSSCCCCCCCCSSCCC
Conf.Score 9754446799856679776899996886455464544434456898489986888868886794557898679997787766668885655789866657 9
           H:Helix; S:Strand; C:Coil
```

C

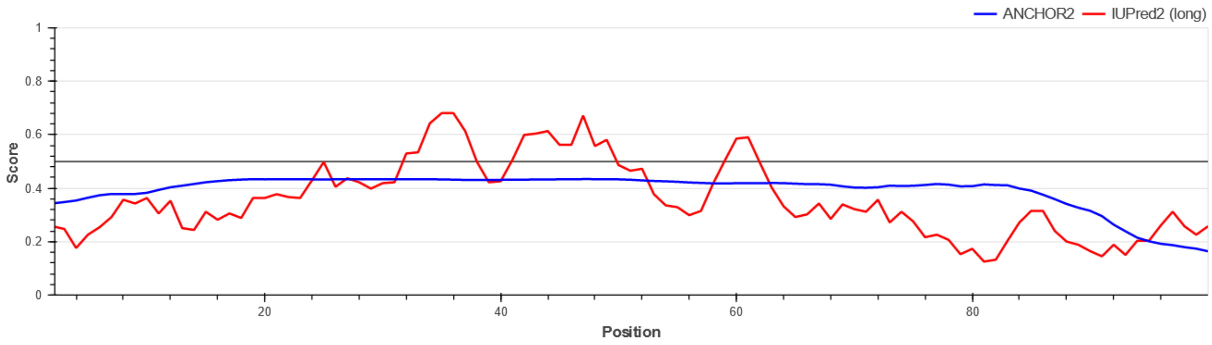

D

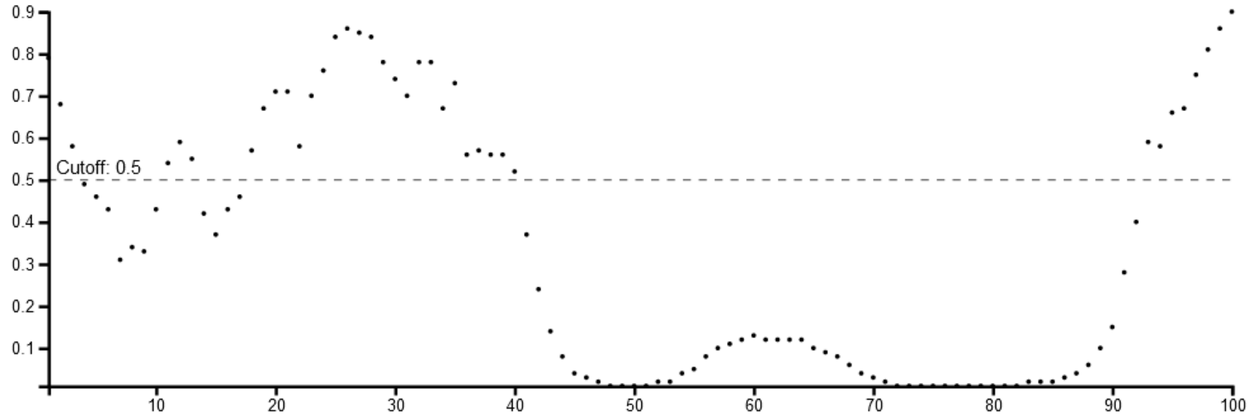

Supplementary Figure S2. Testing of the dimerization activity of CLAMP N-terminal domain using yeast two-hybrid assay. Growth assay plates without histidine are shown (yeasts are unable to grow on this medium in the absence of interaction). AD stands for Activation Domain, BD – for DNA-Binding Domain of GAL4 protein.

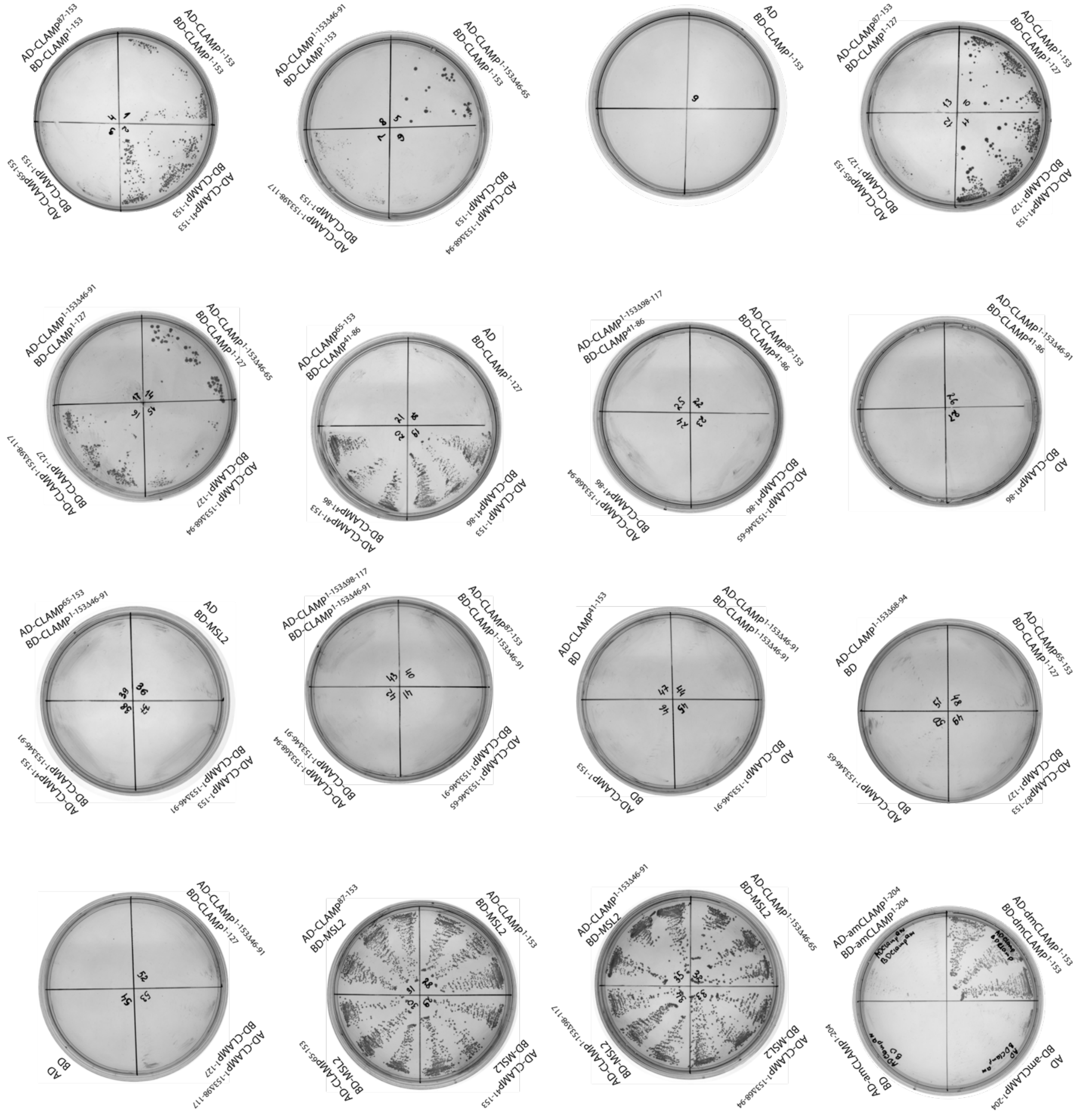

Supplementary Figure S3. Cross-linking of Thioredoxin-tagged CLAMP derivatives using increasing concentrations of glutaraldehyde (GA). Uncropped images.

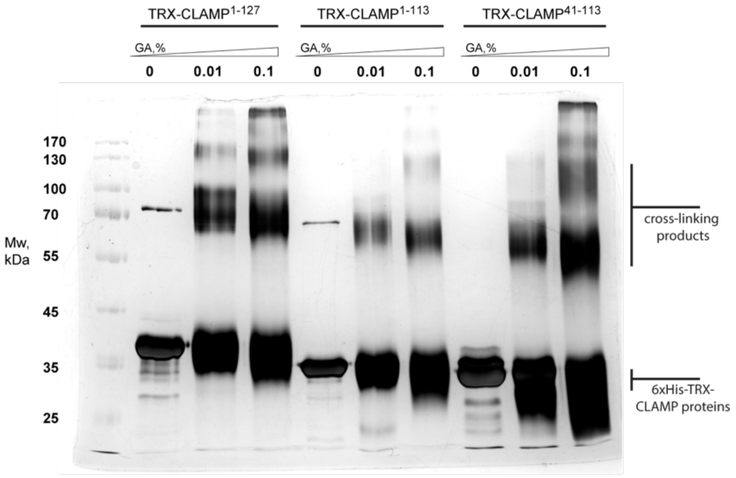

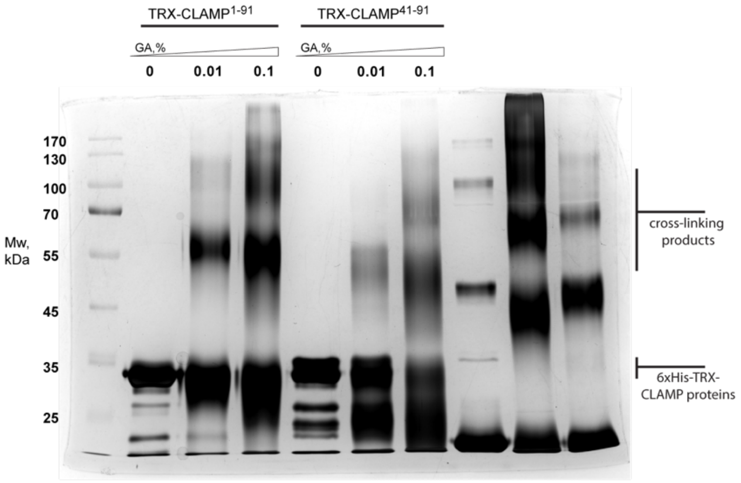

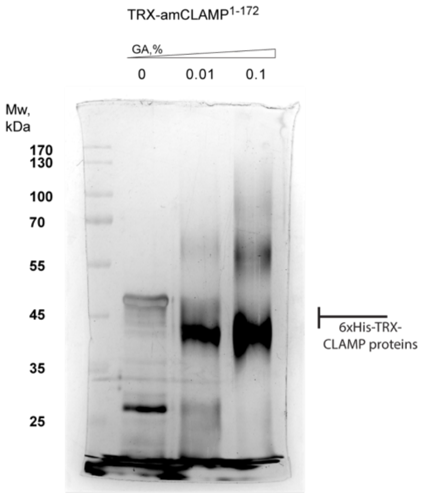

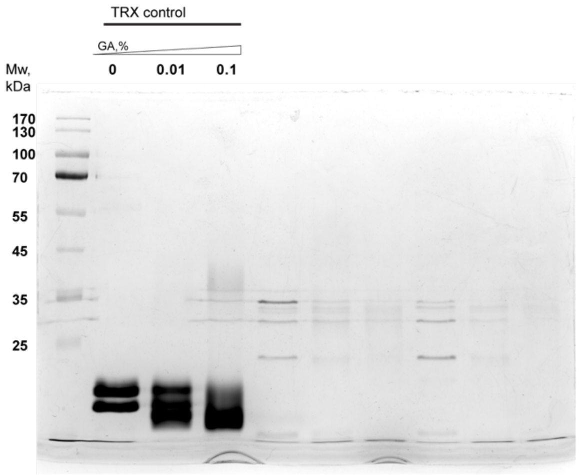

Supplementary Figure S4. Testing of the dimerization specificity of ZADs in GST- and 6xHis- pulldown assays. CLAMP derivatives fused either with GST or with 6xHis-Thioredoxin were co-expressed in bacteria cells and affinity purified with glutathione resin (binds GST-tagged proteins) or with Ni-NTA resin (binds 6xHis-tagged proteins). Co-purified proteins were visualized with SDS-PAGE followed by Coomassie staining. Uncropped images.

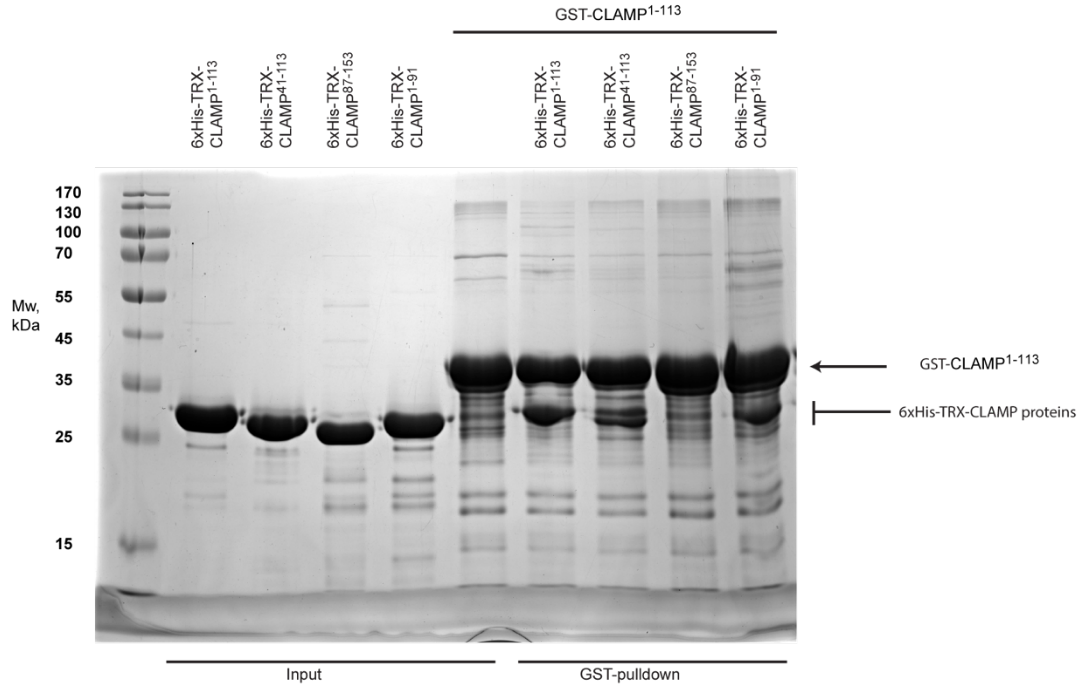

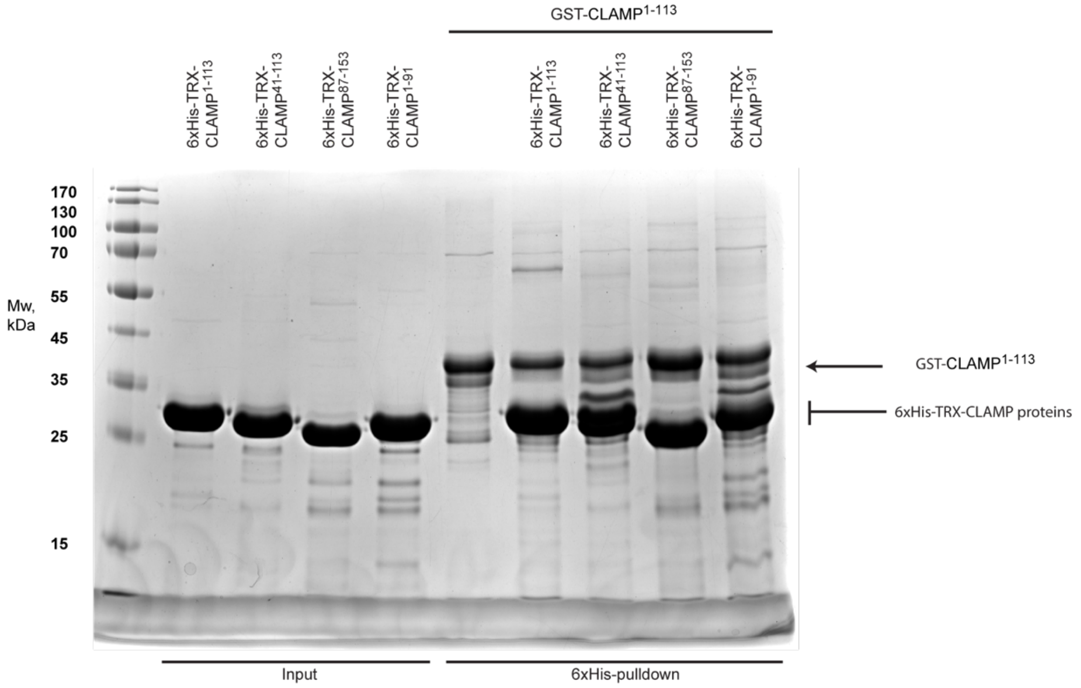

Supplementary Figure S5. ^{15}N-^{1}H-HSQC spectra of *D. melanogaster* CLAMP$^{87–153}$ (blue) and CLAMP$^{41–153}$ (red). The green bracket labels the H_N dispersion of the CLAMP$^{41-86}$ signals.

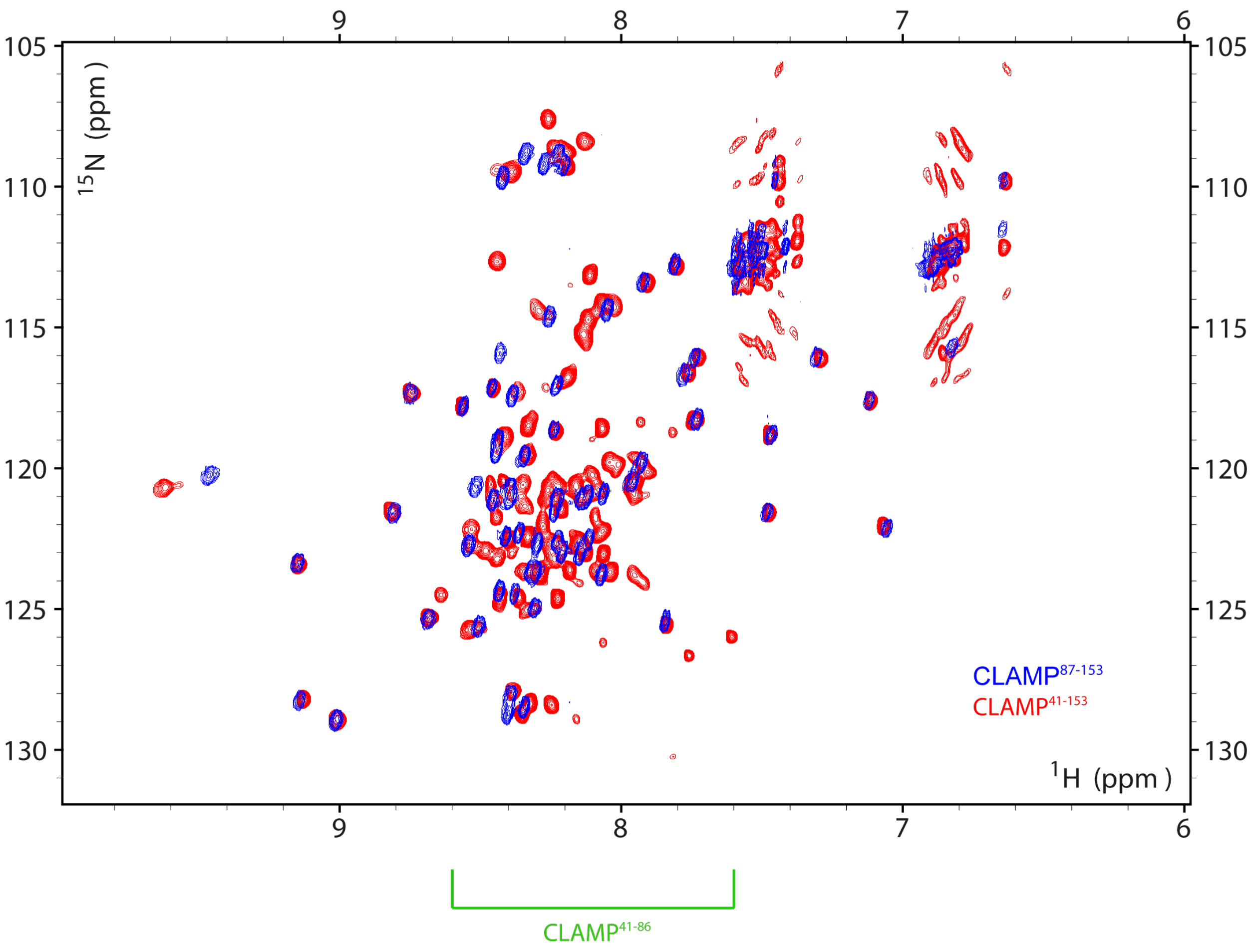

Supplement: Supplementary file 1 [file ijms-23-03862-s001.zip › ijms-1634556-supplementary.pdf]
